# Cadmium Induces Acute Liver Injury by Inhibiting Nrf2 and the Role of NF-κB, NLRP3, and MAPKs Signaling Pathway

**DOI:** 10.3390/ijerph17010138

**Published:** 2019-12-24

**Authors:** Chang Liu, Yaohui Zhu, Zhenxiang Lu, Weina Guo, Bayaer Tumen, Yalan He, Chao Chen, Shanshan Hu, Kangzhi Xu, Yan Wang, Lei Li, Shenghe Li

**Affiliations:** 1 Key Laboratory of Quality & Safety Control for Pork, Ministry of Agriculture and Rural, College of Animal Science, Anhui Science and Technology University, Fengyang 233100, China; liuchang052@126.com (C.L.); ZYH1823539919@163.com (Y.Z.); luzx@ahstu.edu.cn (Z.L.); guown@ahstu.edu.cn (W.G.); heyalan1996@126.com (Y.H.); ccybtx1@163.com (C.C.); ahkshanshan@163.com (S.H.); 15656202506@163.com (K.X.); wy19940210@sohu.com (Y.W.); 2 Shanxi Animal Disease Control Center, Taiyuan 030027, China; tumengbayaer@163.com

**Keywords:** Cadmium, acute liver injury, Nrf2, NLRP3, NF-κB, MAPKs

## Abstract

Acute Cadmium (Cd) exposure usually induces hepatotoxicity. It is well known that oxidative stress and inflammation causes Cd-induced liver injury. However, the effect of nuclear factor erythroid 2-related factor 2 (Nrf2) in Cd-induced liver injury is not completely understood. In this study, we observed Cd-induced liver damage and the potential contribution of Nrf2, nuclear factor-κB (NF-κB), Nod-like receptor 3 (NLRP3), and mitogen-activated protein kinases (MAPKs) signaling pathways. Changes in serum transaminases and proinflammatory cytokines expression showed that Cd could induce acute hepatotoxicity. Moreover, Nrf2 and its downstream heme oxygenase 1 (HO-1) were inhibited by Cd exposure, and Kelch-like ECH-associated protein 1 (Keap1), the inhibitory protein of Nrf2, was increased. Furthermore, NF-κB, NLRP3, and MAPKs signaling pathways were all activated by Cd intoxication. In conclusion, the inhibition of Nrf2, HO-1, and the activation of NF-κB, NLRP3, and MAPKs all contribute to Cd-induced liver injury.

## 1. Introduction

Cadmium (Cd) is a widespread industrial and environmental pollutant, which is has arisen from battery, metals smelting and refining, burning of chemical products, and cigarette smoking. Cd is also a non-essential element that is found in the human body and that of animals. It can be absorbed into the body through the skin, respiratory passages, and digestive tract. The elimination half-life of Cd can be as long as 10 to 30 years [1]. Cd exposure can damage a wide variety of tissues, such as liver, kidney, lung, bone, and brain, and also induce immune, nervous, and reproductive system injuries [2,3,4,5,6]. The liver is the largest gland of both the human and animal body and has multiple physiological functions, such as secretion of bile, participation in substance and energy metabolism, phagocytosis, detoxification, and defense. The complexity and diversity of liver function increases the chances of this gland contacting various toxic factors. Therefore, the liver is more vulnerable to pathogenic factors and, consequently, damage. It was reported that the liver is one of the main target tissues in acute or high dose exposure of Cd [7,8,9]. Cd exposure can also lead to a variety of cancers, especially liver cancer [10].

Cd-induced hepatotoxicity mainly involves in two pathways [8]. Firstly, the initial injury, whereby Cd directly combines with sulfhydryl groups on critical molecules, including glutathione (GSH) and proteins, induces oxidative stress [8]. Second, a subsequent inflammatory injury occurs [8,11]. Although Cd is not a redox reactive metal, its toxicity is mediated via the induction of oxidative stress [11]. Cd binding with sulfhydryl groups results in the generation of reactive oxygen species (ROS) and protein inactivation, and then excessive ROS in turn induces lipid peroxidation and results in DNA damage [12]. The activation of Kupffer cells initiates secondary injury induced by Cd, which results in the production of a large number of proinflammatory cytokines, chemokines, and adhesion molecules to recruit neutrophils to the sites of injury [8]. Kupffer cells and neutrophils both release cytotoxic mediators, including ROS, reactive nitrogen species (RNS), bioactive lipids, and hydrolytic enzymes to cause further liver injuries [13].

Nuclear factor erythroid 2-related factor 2 (Nrf2) is one of the most important transcription factors that initiate a response to oxidative stress. More than 90% of antioxidative genes are regulated by Nrf2. The inhibition of Nrf2 leads to substantial enrichment of ROS. Nod-like receptor 3 (NLRP3) inflammasome, a multiprotein complex, is the most well explored Nod-like receptor. The activated NLRP3 regulates proinflammatory cytokines maturation, such as interleukin-1β (IL-1β) and IL-18, resulting in serious inflammatory damage to the liver [14]. Numerous studies showed that ROS is an essential factor for triggering the activation of NLRP3 inflammasome [15,16,17,18]. Therefore, the inhibition of Nrf2 activity could enhance the generation of ROS to activate NLRP3 inflammasome-induced inflammation [19,20,21]. Furthermore, Cd-induced generation of ROS could activate mitogen-activated protein kinases (MAPKs) signaling pathways, leading to cell death [22]. The suppression or inactivation of Nrf2 resulted in the activation of nuclear factor-κB (NF-κB) mediated transcriptional activity. A previous study suggested that Cd-induced liver injury was much worse in Nrf2-null mice [23]. Meanwhile, another study reported Cd could induce the activation of NLRP3 inflammasome in vascular endothelial cells [24]. However, the effects of Nrf2 and NLRP3 in Cd-induced liver injury have not been fully elucidated. In the present study, we explored the contribution of Nrf2, NF-κB, NLRP3, and MAPKs in Cd-induced liver injury.

## 2. Materials and Methods 

### 2.1. Reagents and Antibodies

CdCl_2_ was obtained from Shanghai Aladdin Biochemical Technology Co., Ltd. (Shanghai, China), with a purity of >98.0%. Alanine/aspartate aminotransferase (ALT/AST) kits were obtained from Nanjing Jiancheng Bioengineering Institute (Nanjing, China). T-PER tissue extraction reagent, NE-PER nuclear and cytoplasmic extraction reagents, SuperSignal West Pico PLUS Substrate kit, and Pierce BCA Protein Assay Kit were obtained from ThermoFisher Scientific (Waltham, MA, USA). Murine IL-1β and IL-6 ELISA kits were purchased from Neobioscience (Shenzhen, China). The antibodies that were used for immunoblotting anti-Keap1, -p38, -phospho-p38, -ERK, -phospho-ERK, -JNK, -phospho-JNK, -NLRP3, -NF-κB p65, and -phospho- NF-κB p65 were purchased from Cell Signaling Technology (Danvers, MA, USA) (1:1000 dilutions). The antibodies against Nrf2 and HO-1 were all purchased from Santa Cruz (Santa Cruz, CA, USA) (all 1:200 dilutions). Antibodies against GAPDH were purchased from Abways Technology (Shanghai, China) (1:2000 dilutions). Peroxidase-conjugated goat anti-rabbit immunoglobulin IgG (H + L), anti-mouse IgG (H + L), and anti-goat IgG (H + L) were obtained from Proteintech Group (Wuhan, China). Other reagents, unless indicated, were obtained from Sigma Chemical Co. (St. Louis, MO, USA).

### 2.2. Animals and Treatments

Specific pathogen-free male ICR mice (20 ± 2 g body weight) were obtained from the Qinglongshan Laboratory Animal Center (Nanjing, China). The mice were fed with a standard laboratory diet and ample water at room temperature with a 12 h light-dark cycle with 60 ± 10% humidity. All of the animals received humane care in line with the institutional animal care guidelines that the Experimental Animal Ethical Committee, Anhui Science and Technology University approved (No.008, Approval date: 25 February 2019).

The mice were randomly separated into two groups: (1) Vehicle control (intraperitoneal injection of saline 0.1 mL/10 g, *n* = 8), or (2) CdCl_2_ (4 mg/kg, *n* = 40), animals were sacrificed after 3 h, 6 h, 12 h, 18 h, and 24 h CdCl_2_ intoxication (eight mice at each time point), then the samples of plasma and liver tissue were collected and stored at −80 °C for further analysis.

### 2.3. Analysis of Serum ALT/AST Activities

The blood samples were collected and then kept at room temperature for 2 h. Serum was harvested after centrifugation at 1500× *g* for 10 min. Serum ALT/AST were measured with kits according to the manufacturer’s protocol.

### 2.4. ELISA Assay

The concentrations of IL-1β and IL-6 in serum were measured with commercial ELISA kits while referring to the manufacturer’s protocols.

### 2.5. Liver Histological Analysis

Tissue slices harvested from the same location within the liver in mice were fixed in 10% phosphate buffered saline-formalin for at least 24 h and all the samples were dehydrated, and then embedded in paraffin for histological check of liver tissue damage. The samples were subsequently sectioned at 5 μm, and then stained with hematoxylin and eosin (H&E) to appraise liver injury. 

### 2.6. Western Blot Analysis

The liver tissue cellular proteins were extracted by using T-PER tissue extraction reagent or NE-PER nuclear and cytoplasmic extraction reagents according to the manufacturer’s protocols. Equal amounts of protein from each sample was separated by 10% SDS-PAGE gel and then transferred to PVDF membrane. After 1 h blocking with 5% BSA at room temperature, the membranes were incubated with primary antibodies at 4 °C overnight. Subsequently, membranes were probed with horseradish peroxidase-conjugated secondary antibodies at room temperature for 1 h. After washing three times with tris-buffered saline-tween (TBST), the membranes were visualized by SuperSignal West Pico PLUS Substrate kit. 

### 2.7. Statistical Analysis

The data were expressed as means ± standard error of mean (SEM). Multiple comparisons among different groups were conducted by one-way analysis of variance (ANOVA) with Dunnett’s post-test, and SPSS 23.0 analyzed all data. *p* < 0.05 was defined as a statistically significant difference. 

## 3. Results

### 3.1. Cadmium Increases the Activities of ALT/AST

**Figure 1:**
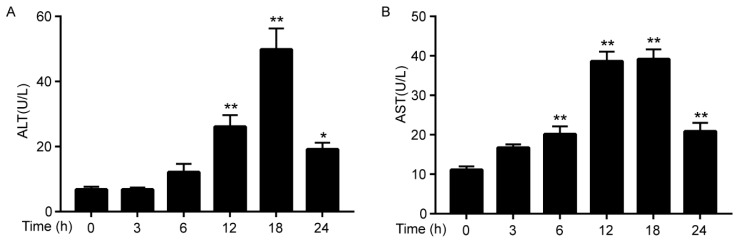
Cd-induced elevated serum Alanine (ALT) (**A**) and aspartate aminotransferase (AST) (**B**) activities in mice. Data were expressed as means ± SEM (*n* = 8). * *p* < 0.05, ** *p* < 0.01 compared to control.

Serum ALT and AST level were measured to evaluate Cd-induced liver injury. As shown in Figure 1, when compared to the control group, the serum ALT level significantly increased after Cd exposure for 12 h and AST level increased significantly after Cd exposure for 6 h, and both ALT and AST concentrations were highest after Cd exposure for 18 h. However, the concentrations of ALT and AST were decreased after Cd exposure for 24 h as compared to 18 h. The results indicated acute Cd intoxication, resulting in liver injury after Cd exposure for 6 h, and damaged liver tissue was gradually recovered after 18 h. 

### 3.2. Cadmium Induces Pro-Inflammatory Cytokines Expression in Serum

**Figure 2:**
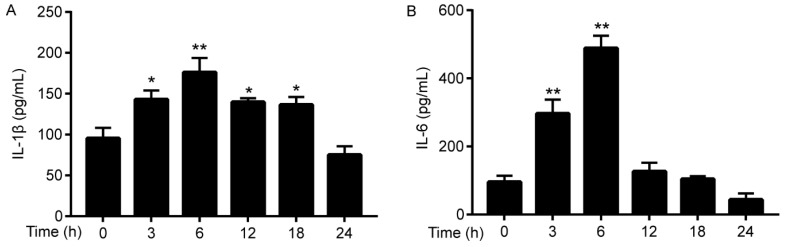
Cd-induced increase in serum inflammatory cytokines IL-1β (**A**) and IL-6 (**B**) in mice. Data were expressed as means ± SEM (*n* = 8). * *p* < 0.05, ** *p* < 0.01 compared to control.

Secondary inflammation plays a vital contribution on Cd-induced liver injury; therefore, ELISAs evaluated proinflammatory cytokines. As shown in Figure 2A,B, the concentration of IL-1β and IL-6 were elevated at all time intervals examined, and the concentrations of the cytokines were greatest after 6 h of Cd intoxication.

### 3.3. Cadmium Induces Histopathological Changes

**Figure 3:**
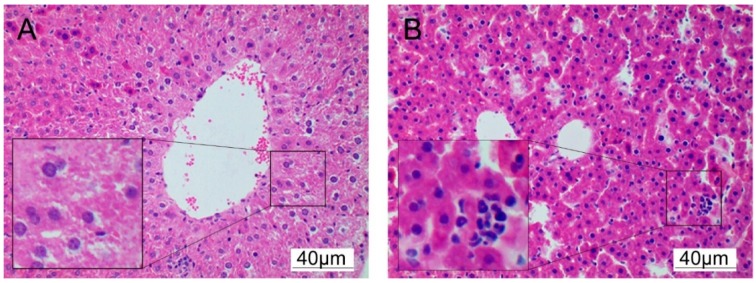
Histological observation of Cd-induced liver injury of mice. After Cd exposure for 18 h, the liver was collected for section and processing for H&E staining (*n* = 3). (**A**) Control, (**B**) Cd (4 mg/kg) 18 h (magnification× 200).

Histopathological examination of liver tissue was used to assess Cd induced hepatotoxicity. After Cd exposure for 18 h, the liver tissue showed extensive necrosis and neutrophil infiltration, as shown in Figure 3.

### 3.4. Cadmium Treatment Results in Nrf2 Inhibition in Liver

**Figure 4:**
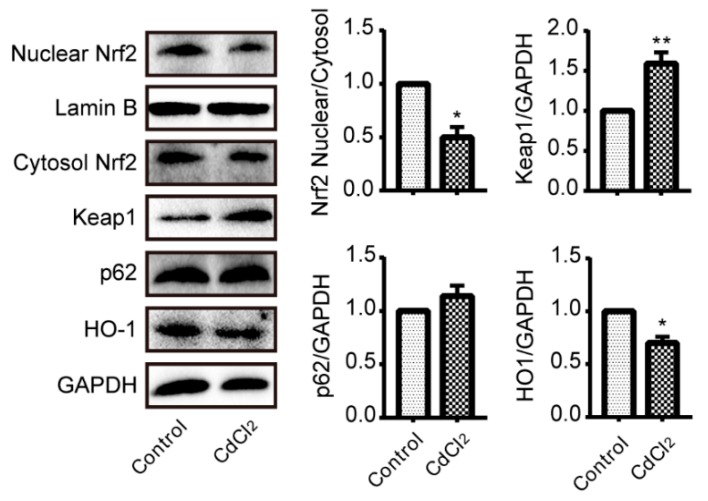
Cd inhibited Nrf2 signaling pathway in mouse liver (*n* = 4). Western blotting analysis of Nrf2, Keap1, p62 and HO-1. The density of the western blotting was normalized for GAPDH/Lamin B. * *p* < 0.05, ** *p* < 0.01 as compared to control.

Nuclear translocation is an essential indicator of Nrf2 activation; therefore western blotting was used to determine the activation of Nrf2. As shown in Figure 4, compared to control group, nuclear translocation was inhibited after CdCl_2_ challenge. Meanwhile, Keap1, which is a negative regulator of Nrf2, was significantly upregulated by CdCl_2_ exposure. Besides, CdCl2 also markedly decreased Nrf2-regulated antioxidative enzyme HO-1. However, Cd has no obvious effect on the expression of p62. 

### 3.5. Cadmium Treatment Results in NF-κB p65, NLRP3, and MAPKs Activation in Liver

**Figure 5:**
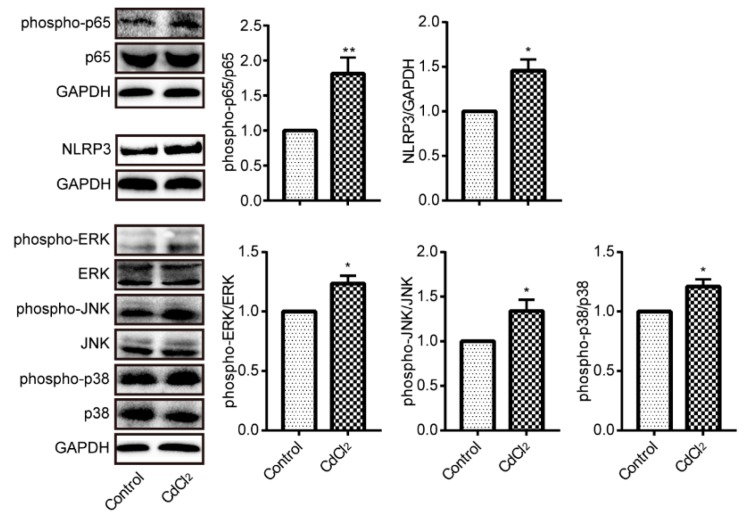
Cd activated nuclear factor-κB (NF-κB), Nod-like receptor 3 (NLRP3), and mitogen-activated protein kinases (MAPKs) in mouse liver (*n* = 4). Phosphorylation of p65, ERK, JNK and p38 and the expression of NLRP3 were evaluated by Western blotting. * *p* < 0.05, ** *p* < 0.01 compared to control.

The phosphorylation of p65 subunit plays a key role in regulating NF-κB activation. Therefore, we detected phosphorylated-p65 by western blotting. Phosphorylation of p65 was increased after CdCl_2_ exposure, as shown in Figure 5. NLRP3 is also a critical molecular in inflammatory injury, thus we evaluated the expression of NLRP3 by western blotting. It was upregulated in the liver when the mice were intoxicated by CdCl_2_. MAPKs is a family of serine/threonine kinases, which are involved in signal transduction pathways that control proliferation, gene expression, differentiation, inflammation, cell survival, and apoptosis [25]. Phosphorylation is required for MAPKs activation, and the results in Figure 5 showed a significant activation of ERK, JNK, and p38. 

## 4. Discussion

The present study demonstrated that Cd exposure significantly damaged mice liver. Firstly, the serum ALT/AST were elevated after Cd treatment, and inflammatory injury was also observed by increased proinflammatory cytokines. Furthermore, Keap1/Nrf2 antioxidative signaling pathway was inhibited and simultaneously proinflammatory signaling pathways of NLRP3, NF-κB, and MAPKs were activated as a result of Cd exposure in mice liver. 

In the present study, serum ALT/AST activities reached a summit after 18 h of Cd intoxicant, indicating acute liver damage; meanwhile, oxidative stress was considered as the initial stimulation of Cd-induced liver injury [8]. Therefore, we choose the 18 h as the time point to measure the activation of Nrf2, which is essential in regulating the expression of antioxidative genes, such as HO-1. As the most important transcription factor in regulating antioxidative genes, Nrf2 was activated when it translocates into the nucleus, heterodimerizes with small Maf preteins, and then binds to the ARE sequence [26], which is known as canonical mechanisms of Nrf2 activation [27]. Our results showed that the expression of Nrf2 in nuclear was decreased after Cd exposure, which is indicative of the inhibition of Nrf2. HO-1, an antioxidative enzyme, which is the downstream of Nrf2, was also suppressed. Keap1, as an adapter subunit of Cullin3-based-E3 ubiquitin ligase, is a negative regulator of Nrf2, which regulates the degradation of Nrf2 [28]. Our data demonstrated that the expression of Keap1 was increased by Cd treatment. The p62, which is another regulator of Nrf2, is involved in non-canonical mechanisms of Nrf2 activation [27]. The role of p62 is mainly sequestering Keap1 to autophagic degradation that ultimately results in the stabilization of Nrf2 [28,29]. However, Cd exposure had no effect on the expression of p62. Taken together, increasing the expression of Keap1 results in the inhibition of Nrf2, which contributes to Cd-induced oxidative injury and they are in line with previous findings [23,30]. 

NF-κB, another protein complex, is a redox-sensitive transcription factor that is involved in the transcription of proinflammatory cytokines. It is well known that excessive ROS can activate the transcription of NF-κB resulting in overproduction of proinflammatory mediators. A previous study demonstrated that inhibition of Nrf2 showed more prominent activation of NF-κB, which is mediated by activation of inhibitor of nuclear factor κB kinase (IKK) and the degradation of NF-κB inhibitor α (IκB-α) [31]. Moreover, the activity of NF-κB p65 subunit also regulates Nrf2 induced antioxidant responsive element (ARE)-linked gene expression [32]. Firstly, increased p65 could promote Keap1 nuclear translocation, which could abrogate Nrf2-ARE signaling [33]. Moreover, phosphorylated p65 displays a preference of binding to CREB-binding protein (CBP), a transcriptional co-activator for Nrf2, which results in limited formation of CBP-Nrf2 complex [23,28,29]. In addition, p65 could prevent Nrf2 heterodimer formation leading to decrease of ARE-mediated gene expression [32,34]. Our results showed that phosphorylated p65 subunit was enhanced and Nrf2-regulated HO-1 expression was particularly decreased in Cd-treated mice liver, which might reveal that the activation of NF-κB contribute to Cd-induced Nrf2 inactivation.

Inflammation was considered as the subsequent injury for Cd-induced hepatotoxicity. NLRP3 inflammasome facilitates a wide range of microbial and oxidative stress responses, and it mediates the cleavage of caspase-1 and the secretion of the proinflammatory cytokine IL-1β, which could induce serious inflammation. Inhibition of Nrf2 leads to overproduce of ROS, which is an essential factor in NLRP3 activation. In the present study, we found NLRP3 was elevated by Cd exposure, which might be relative with suppression of Nrf2 and it is consistent with previous reports [24,35].

MAPKs, which are mainly composed of ERK, JNK, and p38, are critical for regulating oxidative and inflammatory damages. It is well studied that oxidative stress induces phosphorylation of JNK, ERK, and p38 in different cell types [36]. Cd-induced oxidative stress could stimulate MAPKs in different situations. It was reported that Cd could activate ERK, JNK, and p38 in neuronal PC21 and SH-SY5Y cells [37,38]. However, only ERK and JNK were activated in Cd-treated human renal endothelial cells [39]. Another study showed ERK was the major pathway involved in Cd-treated prostate epithelial cells [40]. Moreover, Zou et al. [41] reported that Cd treatment resulted in the activation of MAPKs, while ERK and p38 inhibitors, but not JNK inhibitor, attenuated Cd-induced hepatotoxicity. In this study, we found that ERK, JNK, and p38 were all activated by Cd, which contributes to Cd-induced hepatotoxicity. Therefore, the findings revealed that Cd-induced MAPKs activation is likely to be specific to the cell type and dose tested. Furthermore, the MAPKs are implicated to be able to activate IKK to induce NF-κB nucleus translocation and activate gene transcription [42], which suggested that Cd-induced MAPKs activation might be contribute to Cd-induced NF-κB activation.

## 5. Conclusions

The results of our study suggest that the suppression of Nrf2 and its downstream HO-1 greatly contributed to Cd-induced acute liver injury, and NF-κB, NLRP3, and MAPKs were all involved in Cd-induced hepatotoxicity. Nrf2, NF-κB, NLRP3 and MAPKs may be therapy targets for amelioration of Cd-induced liver damage. However, numerous signaling pathways are involved in the regulation of Nrf2 activation, including canonical and non-canonical mechanisms of Nrf2 activation; therefore, more investigation should be explored in the Cd-regulated Nrf2 signaling pathway.
